# Review of bacterial and viral zoonotic infections transmitted by dogs

**Published:** 2015

**Authors:** I Ghasemzadeh, SH Namazi

**Affiliations:** * Research center for Infectious and Tropical Disease, Hormozgan University of Medical Sciences, Bandar Abbas, Iran; ** Student Research Committee, Hormozgan University of Medical Sciences, Bandar Abbas, Iran

**Keywords:** bacterial and zoonotic infections, viral infections, dogs, rabies, noroviruses

## Abstract

Dogs are a major reservoir for zoonotic infections. Dogs transmit several viral and bacterial diseases to humans. Zoonotic diseases can be transmitted to human by infected saliva, aerosols, contaminated urine or feces and direct contact with the dog. Viral infections such as rabies and norovirus and bacterial infections including Pasteurella, Salmonella, Brucella, Yersinia enterocolitica, Campylobacter, Capnocytophaga, Bordetella bronchiseptica, Coxiella burnetii, Leptospira, Staphylococcus intermedius and Methicillin resistance staphylococcus aureus are the most common viral and bacterial zoonotic infections transmitted to humans by dogs. This review, focused on the mentioned infectious diseases by describing general information, signs and symptoms, transmission ways, prevention and treatment of the infection. As far as the infections are concerned, the increase of the knowledge and the awareness of dog owners and the general population regarding zoonotic infections could significantly mitigate zoonoses transmission and consequently their fatal complications.

## Introduction

It is estimated that over 60% of the western families own a pet. The majority of these households keep a dog. Dogs have been kept as pets for over 14 centuries. Many studies have confirmed the precious roles of pets in the human life. Evidence has shown that owning a pet can increase the activity of pet owners and consequently reduced serum cholesterol, low triglyceride levels, and fewer cardiovascular events [**1**,**2**]. Also, some other studies demonstrated that pet owners suffer from depression and mental stress less and have a higher self esteem compared to others. Although dogs have several positive effects on the psychosocial and psychical health of their owners, many diseases among humans are attributed to them [**3**]. Children and immunocompromised individuals are especially at an increased risk of developing zoonoses infections. Several studies demonstrated that domestic dogs have a dramatic role in developing zoonoses disease and hospitalization [**4**,**5**].

Regarding domestic dogs, the increase in the population of stray and semi domestic dogs in urban areas has increased the risk of zoonoses diseases. About 5 million people throughout the world are annually bitten by dogs. Many parasitic and zoonotic pathogens are transmitted by dogs [**6**,**7**]. This review focused on the most important viral and bacterial zoonotic diseases, which can be transmitted by dogs.

**Rabies**

Rabies is a single strand RNA virus belonging to the Rhabdoviridae family. Rabies infection is an ancient disease with a high mortality rate in human and animal population. Based on the World Health Organization reports, annually between 30000 and 70000 deaths occurred throughout the world due to rabies infection [**8**]. Dogs are the major animal reservoirs for rabies infection. The majority of the infected patients in developing countries are infected by dog bites while, in developed countries, wild animals including raccoons, bats and foxes are the main cause for rabies transmission [**9**]. In a study in the United States, a rabies control program was conducted by using extensive vaccination in domestic dogs and reducing the rabies infection [**8**]. The incubation period for rabies varies between 4 days to several years depending on the location of the inoculating wound and the amount of induced viruses. Patients may present agitation, anxiety, confusion, hallucination, and hydrophobia. Post exposure prophylaxis with frequent doses of human rabies immunoglobulin (HRIG) within 14 days after the suspected dog bite can prevent the disease. Washing the wound with water and liquid soap can dramatically reduce the viral lead and consequently the probability of rabies infection [**10**]. 

**Noroviruses**

Noroviruses are a heterogeneous single strand RNA virus belonging to the Caliciviridae family. Noroviruses are the main cause of sporadic and epidemic gastroenteritis in humans [**11**]. This virus can affect humans of all ages. The virus can be found in the gastrointestinal tract and consequently in the feces or diarrhea of the infected dogs. It can be transmitted from contaminated food or water to humans and the infection can rapidly spread in the human population by fecal oral rate. Serum therapy should be considered for patients with acute gastroenteritis [**12**].

**Pasteurella**

Pasteurella species are Gram-negative coccobacilli, which were primarily found in animals. Pasteurella spp are normal flora of the upper respiratory tract of dogs and cats. Pasteurella infection can be transmitted to humans by direct and indirect contact such as dog or cat bites or licks and even cat scratches [**6**]. Several infectious diseases in humans are attributed to Pasteurella spp. The soft tissue infection is the most important infection transmitted by Pasteurella spp. However, meningitis, bone and joint infections and respiratory infection can be transmitted by Pasteurella spp [**13**]. In a prospective study in United States, the author demonstrated that Pasteurella spp. was the most frequent organism isolated from dog and cat bites [**2**]. Pasteurella infection can be treated by second and third generation cephalosporin, macrolides, fluoroquinolones, cotrimoxazole, and penicillin [**14**].

**Salmonella**

Salmonella species are anaerobic and motile gram-negative bacilli that colonize in the large intestine of a variety of mammals, especially in the distal part of the colon and the mesenteric lymph nodes of the canine. Humans can also get infected through the gastrointestinal tract [fecal transmission] and develop several infectious diseases such as gastroenteritis, enteric fever, bacteremia and osteomyelitis. Gastrointestinal diseases are the most prevalent clinical presentations of salmonella in human and dogs; however, the majority of infected animals or humans is asymptomatic and may shed the pathogen through feces for a period of 6 weeks and transmit the pathogen to other animals or individuals. In developing nations, Salmonella spp. is also more prevalent than in developed countries [**15**,**16**]. An antibiogram should be considered for patients infected with Salmonella spp. however, it could be treated by various families of antibiotics including fluoroquinolones, beta-lactams, and macrolides [**17**].

**Brucella**

Brucellosis is one of the most prevalent zoonoses, which imposes a heavy burden on the national health services. It is commonly transmitted to humans by consuming unpasteurized dairy products. Various types of brucella spp. have been recognized; that resulted in human brucellosis such as B. melitensis, B. abortus and B. suis but, B. canis has been less known as an usual pathogen in brucellosis infection in humans [**18**,**19**]. Although B. canis is not responsible for the brucellosis infection in humans, the reported cases were more often seen among farmer populations who had a history of exposure to body fluids of dogs, which were infected with B. canis. The incubation period may last for one to four weeks up to several months [**19**]. The patients may be asymptomatic or may even present serious clinical symptoms especially fever, night sweats and low back pain in the endemic region that should be differentiated from tuberculosis and other malignancies [**20**]. Brucellosis should be treated in order to avoid complications and sequelae of the disease. Combination therapies, which are widely employed in the treatment of brucellosis, consisted of doxycycline plus streptomycin or rifampin for 6 weeks [**21**].

**Yersinia enterocolitica**

Y. enterocolitica is a gram-negative coccobacillus zoonotic pathogen that causes yersiniosis in human and animals. Several animals are main reservoirs for Y. enterocolitica including birds, pigs, deer, and cattle. The pathogen has been isolated from dog bite wound in some studies [**22**]. The patients may be asymptomatic in early stage and when the pathogen invades the mucosal surface of the intestine, watery or bloody diarrhea may be present. The pathogen can also involve the peyer’s patches and represent the appendicitis symptoms [**23**,**24**]. Y. enterocolitica is mostly a self-limiting disease that does not need antibiotic therapy, however, patients with severe infection and immunocompromised patients should be treated with a combination of an aminoglycoside and doxycycline [**24**]. 

**Campylobacter**

Campylobacter spp. including campylobacter jejuni and campylobacter coli are gram-negative bacteria that usually result in campylobacter enteritis. This organism normally lives in the gastrointestinal tract of many animals. Direct contact with infected animals or their products is a leading cause of campylobacter transmission. Dogs and puppies are the major reservoirs for campylobacter. For example, in a study it was demonstrated that about 47% of the fecal specimens of dogs’ campylobacter was isolated [**25**,**26**]. The incubation period in campylobacter enteritis varies from one to seven days. Most of the patients present fever, vomiting, diarrhea, and abdominal pain. Also, bloody diarrhea may be present in more than 50 percent of the infected patients. Convulsion and seizure may be observed in some patients [**27**]. This infection is usually self-limited and does not need antimicrobial therapy. Focus on correction of electrolyte imbalance and hydration should be considered. Antibiotic therapy with fluoroquinolones, macrolides, or aminoglycosides is indicated in patients with severe disease [**28**].

**Capnocytophaga**

Capnocytophaga canimorsus is a gram-negative bacterium, which is found in the normal flora of the oropharyngeal tract of dogs and cats. The pathogen is mostly transmitted to human by dogs bite and causes an overwhelming sepsis, particularly in elderly, immunocompromised or asplenic patients [**25**]. The pathogen can also lead to other fatal infections including meningitis, osteomyelitis, arthritis, lung abscess or empyema and endocarditis. In addition, thrombotic thrombocytopenic purpura and hemolytic uremic syndrome can be associated with capnocytophaga septicemia especially in immunocompromised patients [**25**,**29**]. The literature data have demonstrated that the mortality rate due to capnocytophaga septicemia is estimated to be of one third of the infected patients. Accordingly, early empirical therapy with third generation cephalosporins in patients who received a dog bite should be considered [**30**].

**Bordetella bronchiseptica**

Bordetella bronchiseptica is a gram-negative rod bacterium belonging to the genus Bordetella. The pathogen normally lives in the upper respiratory tract of the mammals such as dogs and cats and is transmitted to humans by aerosol. B. bronchiseptica can lead to acute tracheobronchitis in dogs, which presents with harsh and kennel cough [**31**,**32**]. Human infection with B. bronchiseptica is very rare; however, the pathogen can also cause pneumonia and upper respiratory tract infection in dog owners [**33**]. Evidences demonstrated that this organism is resistant to macrolides and cephalosporins; however, in several studies, the organism was sensitive to fluoroquinolones and Trimethoprim/ sulfamethoxazole [**34**].

**Coxiella burnetii**

C. burnetii is an obligate intracellular gram-negative bacterium that causes Q fever in humans. The pathogen normally infects individuals via aerosol and direct contact with the body fluids of the infected animals. Although dogs are not the main reservoirs for C. burnetii, however, in a study it was demonstrated that C. burnetii was isolated from approximately 10 percent of farm dogs [**35**]. In addition, in another study by Buhariwalla and colleagues, it was reported that C. burnetii could be transmitted to human from an infected parturient dog. In addition, the patients developed the symptoms of Q fever including fever, chills, nausea, vomiting and productive cough. Opacity is a common finding in chest radiography, and, in physical examination, crackles may be heard during auscultation. The incubation period in this study was estimated to be between 8 and 12 days after the exposure to the infected animal. The patients with C. burnetii can be treated with fluoroquinolones or doxycycline successfully [**36**]. 

**Leptospira**

L. interrogans is an aerobic spirochete, which is the major cause of Leptospirosis in human. Leptospirosis is worldwide zoonoses that are mostly transmitted to human by environmental sources including contaminated soil, water, urine, or tissue of the infected animals. Rodents are the major reservoirs for Leptospirosis; however, domestic animals including dogs can play an important role in leptospirosis transmission in endemic regions [**37**]. Mucosal surfaces of the human body including eye, vagina, nose, mouth, or erosive lesions, which have a direct contact with the contaminated urine, are the main ways of Leptospirosis transmission. The incubation period for this infection is averagely of about 10 days (ranging from 2 to 26 days) [**38**,**39**]. Leptospirosis may present with a variety of symptoms from no symptom to fever, nonproductive cough, headache, musculoskeletal pain, diarrhea, nausea, vomiting, alveolar hemorrhage, and even meningitis [**39**]. Several antibiotics such as doxycycline, ceftriaxone, cefotaxime, penicillin, amoxicillin, and ampicilin have been successfully employed for the treatment of Leptospirosis [**40**].

**Staphylococcus intermedius**

S. intermedius is a gram-positive bacterium with a coagulase activity that normally lives in the anterior part of the nasal cavity of several animals such as dogs, pigeons, and horses. Some evidences demonstrated that this pathogen could also be isolated from the gingival of healthy dogs [**41**]. S. intermedius is not a common zoonotic pathogen in humans; however, several studies demonstrated that this bacterium is a potential pathogen associated with dog bite wounds and cellulitis can develop in inflicted humans [**42**,**43**]. This pathogen should be discriminated from staphylococcus aureus. Penicillin and amoxicillin-clavulanate are effective in the treatment of this infection [**44**].

**Methicillin resistance staphylococcus aureus**

Methicillin resistance staphylococcus aureus (MRSA) is a major cause of fatal infection in humans. Several investigations have reported that this pathogen has been isolated from some animals such as pigs, horses, cattle, cats and dogs. Of them, some believed that companion animals were the main reservoirs for the transmission of MRSA, being able to transmit the bacterium by direct contact with their owners. However, it seems that animal to human infection of MRSA is more seen in immunocompromised patients. Nevertheless, some evidences showed that this bacterium could be transmitted to healthy humans who own an infected animal [**45**,**46**]. Traditional anti staphylococcal antibiotics are not more effective in the treatment of infections caused by MRSA. Accordingly, newer drugs including vancomycin, linezolid and daptomycin are widely used in the treatment of MRSA infections [**47**].

## Conclusion

Zoonoses are diseases that implicate both humans and animals and can be transmitted either by domestic pets or by wildlife animals. Many animals and their products can be reservoirs of zoonoses pathogens. Among them, dogs are responsible for the transmission of several zoonotic diseases to their owners. Thus, dog owners should be informed regarding the zoonotic diseases and their ways of transmission to reduce these infections in human population. Several prophylactic and therapeutic strategies have been introduced in order to decrease the zoonotic diseases. Dog owners are recommended to wash their hands after any direct contact with their dogs, their products, urine, or feces. Most of the viral and bacterial infections are transmitted from dogs to humans by dog bite; however, other infections caused by protozoa have a fecal oral transmission. Thus, food hygiene such as washing vegetables well and cooking meats adequately should be carefully done in order to eliminate the rate of zoonotic infections. 

In addition, dogs should also be treated for diarrheal infections. Moreover, dog owners should feed their dogs with cooked meat to prevent campylobacter and salmonella infections. Raw meat and eggs should not be fed to dogs due to higher rate of infection susceptibility. Rabies vaccination should be considered for domestic dogs and the dog owners should also be aware of benefits of rabies vaccination before and after dogs bites. Many authors reported that increasing the knowledge of dog owners regarding dog associated zoonotic infections and prevention strategies can dramatically reduce the zoonotic infections in dog owners and their families.
